# A Review on the Role of miR-1246 in the Pathoetiology of Different Cancers

**DOI:** 10.3389/fmolb.2021.771835

**Published:** 2022-01-03

**Authors:** Soudeh Ghafouri-Fard, Tayyebeh Khoshbakht, Bashdar Mahmud Hussen, Mohammad Taheri, Mohammad Samadian

**Affiliations:** ^1^ Department of Medical Genetics, School of Medicine, Shahid Beheshti University of Medical Sciences, Tehran, Iran; ^2^ Men’s Health and Reproductive Health Research Center, Shahid Beheshti University of Medical Sciences, Tehran, Iran; ^3^ Department of Pharmacognosy, College of Pharmacy, Hawler Medical University, Erbil, Iraq; ^4^ Center of Research and Strategic Studies, Lebanese French University, Erbil, Iraq; ^5^ Institute of Human Genetics, Jena University Hospital, Jena, Germany; ^6^ Skull Base Research Center, Loghman Hakim Hospital, Shahid Beheshti University of Medical Sciences, Tehran, Iran

**Keywords:** miRNA, MiR-1246, cancer, expression, biomarker, *in vivo*, *in vitro*, diagnosis

## Abstract

miR-1246 is a microRNA firstly recognized through application of a high throughput sequencing technique in human embryonic stem cells. Subsequent studies have shown the role of this microRNA in the carcinogenesis. miR-1246 has been found to exert oncogenic roles in colorectal, breast, renal, oral, laryngeal, pancreatic and ovarian cancers as well as melanoma and glioma. In lung, cervical and liver cancers, studies have reported contradictory results regarding the role of miR-1246. miR-1246 has been reported to regulate activity of RAF/MEK/ERK, GSK3β, Wnt/β-catenin, JAK/STAT, PI3K/AKT, THBS2/MMP and NOTCH2 pathways. In addition to affecting cell cycle progression and proliferation, miR-1246 can influence stemness and resistance of cancer cells to therapeutics. In the current review, we describe the summary of *in vitro* and *in vivo* studies about the influence of miR-1246 in carcinogenesis in addition to studies that measured expression levels of miR-1246 in clinical samples.

## Introduction

miR-1246 has been firstly recognized through application of a high throughput sequencing technique in human embryonic stem cells (Morin et al., 2008). Subsequent studies have mapped the human miR-1246-coding gene, i.e., *MIR1246* gene on chromosome 2q31.1 and reported the impact of p53 on the regulation of its expression (Zhang et al., 2011). Notably, the nucleotide sequence of the mature miR-1246 is identical to the central region of the RNU2-1 RNA (Xu et al., 2019), a small nuclear RNA which constructs the scaffold for establishment of the U2 complex in the spliceosome (Patel and Bellini, 2008).

Theoretically, the stem-loop TaqMan technique for detection of miR-1246 is expected to amplify both miR-1246 and RNA, U2 Small Nuclear 1 (RNU2-1). However, the poly-A tailing SYBR strategy can differentiate between miR-1246 and RNU2-1, since the sizes of the amplified fragments can be differentiated through assessment of their meting curves (Xu et al., 2019). Application of the latter strategy for assessment of miR-1246 expression in wild type and *MIR1246* knockout pancreatic adenocarcinoma cells and exosomes originated from these cells has led to identification of a variant of the mature miR-1246 in exosomes that is transcribed from cellular RNU2-1 in an independent manner from Drosha and Dicer miRNA processing enzymes (Xu et al., 2019).

Several researchers have assessed expression of miR-1246 in different cancer cell lines using a variety of miRNA-profiling assays. Subsequently, they have performed functional assays to find the effects of miR-1246 up-regulation or silencing on proliferation and invasive properties of these cells. Finally, the impact of this miRNA on tumor growth has been appraised in xenograft models constructed by injection of human cancer cell lines. In the current review, we describe the summary of these two types of studies in addition to those measured expression levels of miR-1246 in clinical samples.

## Cell Line Studies

Experiments in colorectal cancer cell lines have shown oncogenic role of miR-1246. In this type of cancer, the m (6) A methyltransferase METTL3 oncogene has been shown to increase methylation of pri-miR-1246 to enhance maturation of pri-miR-1246. Notably, miR-1246 has been predicted to suppress expression of the Sprouty Related EVH1 Domain Containing 2 (SPRED2) tumor suppressor, thus increasing activity of MAPK pathway (Peng et al., 2019).

Expression of miR-1246 has been found to be increased in exosomes derived from colorectal cancer cells infected with *Fusobacterium nucleatum*. In fact, this cancer-associated bacterium can enhance pro-metastatic behaviors through delivery of these exosomes into un-infected cells (Guo et al., 2021).

Expression of miR-1246 has also been reported to be surged in SW620, SW480, HCT116, HT29 and LOVO colorectal cancer cells, parallel with down-regulation of Cyclin G2 (CycG2). Experiments in HCT-116 and LOVO cells have verified CycG2 as the target of miR-1246. Up-regulation of miR-1246 has exerted pro-proliferative and pro-invasive effects in these cells, while its silencing has reversed these effects (Wang et al., 2016).

Exosomal and cellular levels of miR-1246 have been reported to be higher in organoid lines generated from colorectal cancer compared with organoid lines from colorectal adenomas. Consistent with this finding, miR-1246 up-regulation and down-regulation have enhanced reduced proliferation of an adenocarcinoma cell line, respectively (Nagai et al., 2021).

Another experiment in breast cancer cells has demonstrated high levels of miR-1246 in metastatic breast cancer cells compared with both non-metastatic cancer cells and non-neoplastic breast cells. miR-1246-containing exosomes from metastatic breast cancer cells can alter viability, migratory potential and chemoresistant phenotype of non-malignant breast cells. Functionally, miR-1246 suppresses expression of Cyclin G2 (Li et al., 2017).

In renal cell carcinoma cells, miR-1246 has an oncogenic effect through suppressing expression of PCK1. Notably, the tumor suppressor long non-coding RNA (lncRNA) GABPB1-AS1 has been shown to sponge miR-1246 in these cells (Gao et al., 2020).

**FIGURE 1:**
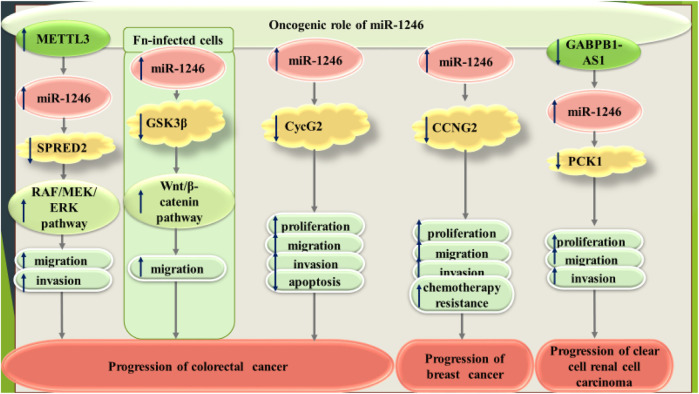
Oncogenic role of miR-1246 in colorectal, breast and renal cancers.

Figure 1 shows the oncogenic role of miR-1246 in colorectal, breast and renal cancers.

miR-1246 has been demonstrated to increase the migration and invasive aptitudes of A549 adenocarcinomic human alveolar basal epithelial cells. In addition, miR-1246 could enhance epithelial-mesenchymal transition (EMT) of lung cancer cells. This miRNA could decrease levels of E-cadherin, while enhancing vimentin and TGF-β levels. Functionally, miR-1246 can target 3′-untranslated region of GSK-3β, thus regulating activity of Wnt/β-catenin pathway (Yang et al., 2019).

Yuan et al. have investigated the impact of ionizing radiation (IR)-induced extracellular miRNAs on proliferation and radioresistance of A549 adenocarcinomic cells. They have reported particular abundance of miR-1246 outside of cells compared with its levels inside the cells. Irradiation could increase expression levels of miR-1246 in A549 and H446 cells in dose- and time-dependent manners. Extracellular miR-1246 has been shown to be transferred from donor cells to recipients through a non-exosome associated route enhancing proliferation and resistance of A549 cells to irradiation. Functionally, miR-1246 reduces expression of death receptor 5 (DR5) (Yuan et al., 2016).

miR-1246 has been among up-regulated miRNAs in the sphere-forming cells compared with the parental A549 and HCC1588 cells. Suppresion of miR-1246 has led to reduction of levels stemness and EMT markers in these cells. Moreover, anti-miR-1246 could suppress proliferation, sphere-formation, colony forming ability and invasiveness of lung cancer cells (Kim et al., 2016). Similarly, Huang et al. have reported up-regulation of miR-1246 and METTL3 in A549 and H1299 cells, parallel with down-regulation of PEG3. METTL3 has been shown to affect m6A marks of miR-1246, therefore increasing expression of miR-1246. Cumulatively, m6A methyltransferase METTL3 modifies the m6A marks of miR-1246 to up-regulates miR-1246 and subsequently increase progression of lung cancer (Huang et al., 2021).

**FIGURE 2:**
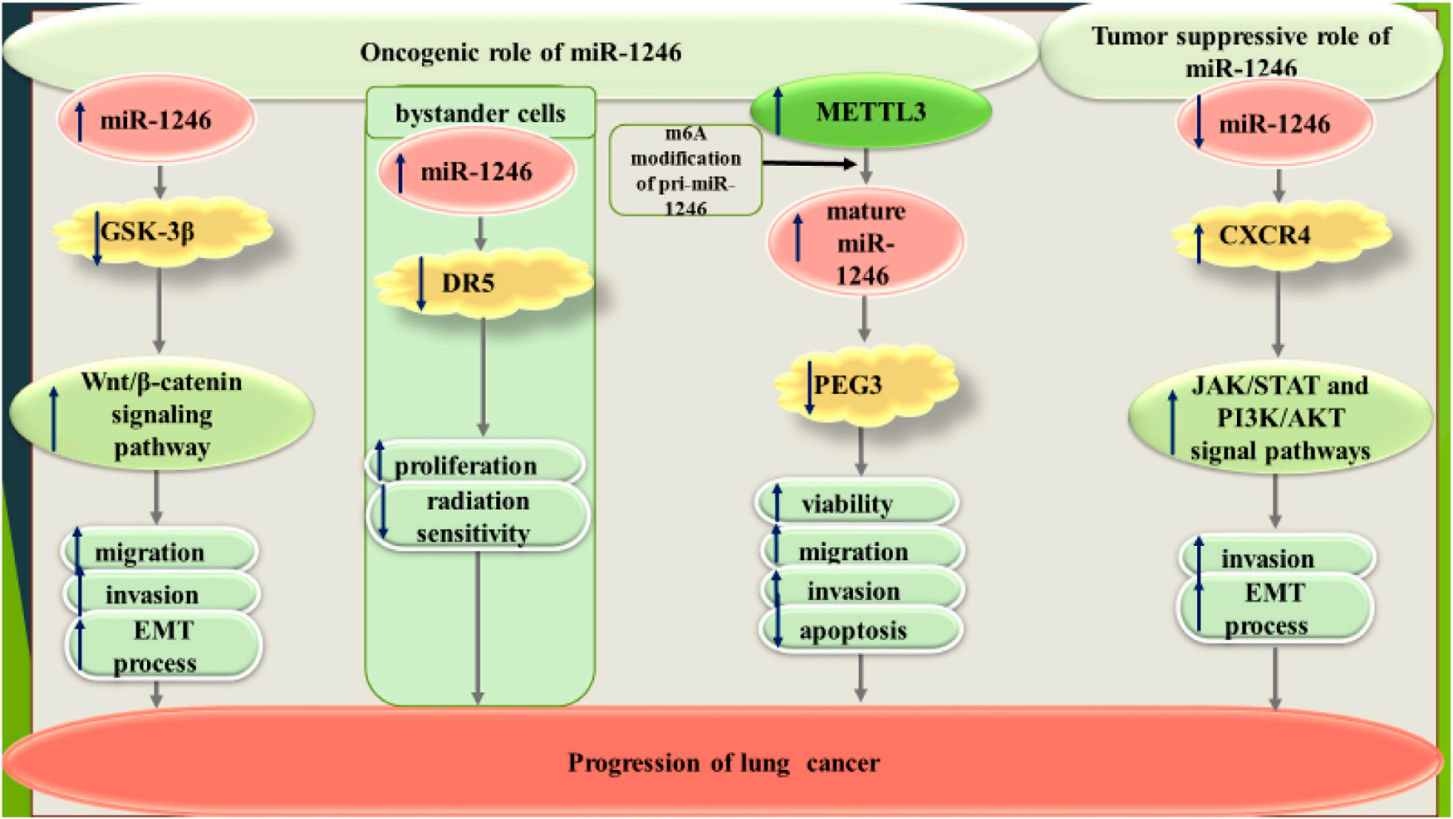
Dual roles of miR-1246 in lung cancer.

Contrary to these studies, Xu et al. have reported down-regulation of miR-1246 in A549, H1650 and H1299 cell lines compared to a normal human bronchial epithelial cell line. MiR-1246 overexpression remarkably inhibited cell invasion as well as up-regulated E-cadherin expression and down-regulated N-cadherin, Vimentin, ZEB1 and Snail expressions in A549 cells. Further studies have confirmed CXCR4 as a target gene of miR-1246, and CXCR4 silence significantly abolished the promotion effect of miR-1246 suppression on cell invasion and EMT process in A549 cells. Besides, miR-1246 blocked JAK/STAT and PI3K/AKT signal pathways by regulation of CXCR4 (Xu et al., 2018). Figure 2 shows dual roles of miR-1246 in lung cancer.

**FIGURE 3:**
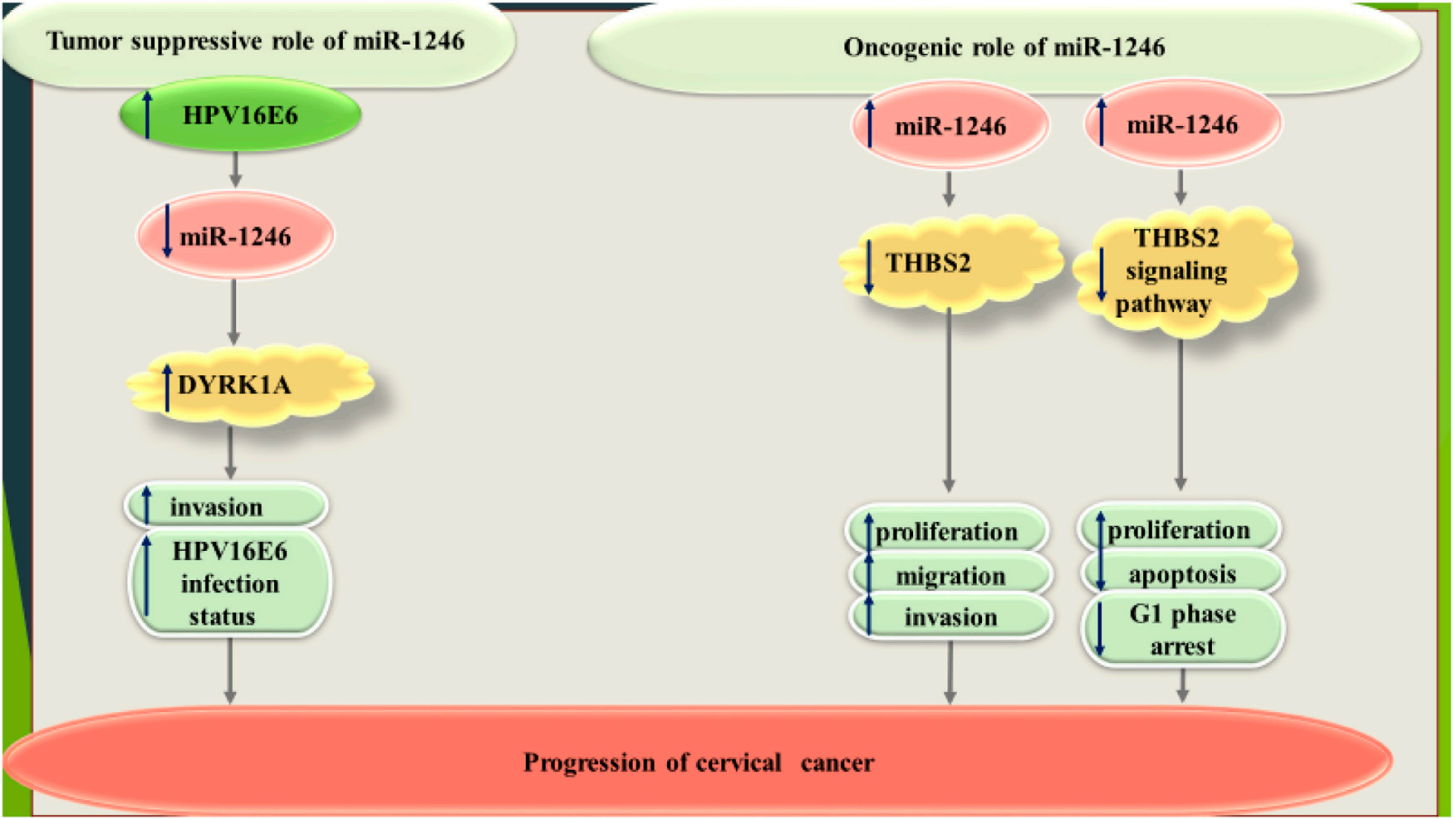
Dual roles of miR-1246 in cervical cancer.

In SiHa HPV16-positive cervical cancer cell line, HPV16 E6 silencing has led to enhancement of miR-1246 expression, thus down-regulation of miR-1246 target DYRK1A. Meanwhile, overexpression of HPV16 E6 in HPV-negative C33A cell line has resulted in down-regulation of miR-1246 (Yang et al., 2015). Another study has shown that miR-1246 increases proliferation, invasiveness and migratory potential of SiHa cells through inhibition of expression of thrombospondin 2 (Chen et al., 2014). miR-1246 has also been among up-regulated miRNAs in radioresistant cervical cancer cells. Expression of this miRNA could be enhanced by irradiation of cervical cancer cells. Up-regulation of miR-1246 has increased survival of cervical cancer cells upon irradiation (Zhang et al., 2013). Figure 3 shows dual roles of miR-1246 in cervical cancer.

Experiments in a co-culture model of hepatic stellate cells (HSCs) and hepatocellular carcinoma cells have shown that expression of miR-1246 is activated by HSCs. miR-1246 has been shown to target RORα. Up-regulation of miR-1246 or silencing of RORα has promoted proliferation, invasive properties, and metastatic aptitude of hepatocellular cancer cells through activation of Wnt/β-catenin pathway and enhancement of EMT (Huang J.-L. et al., 2020). Another study has shown that miR-1246 increases invasiveness of hepatocellular carcinoma cells via modulation of CADM1 expression (Sun et al., 2014). Moreover, miR-1246 has been reported to promote stemness features such as self-renewal, resistance to therapeutics, tumorigenic potential, and metastasis through enhancing activity of Wnt/β-catenin pathway. This effect is mediated through down-regulation of expression levels of AXIN2 and GSK3β. Oct4 has been identified as the direct regulator of miR-1246 expression which activates β-catenin in hepatic cancer stem cells (Chai et al., 2016).

**FIGURE 4:**
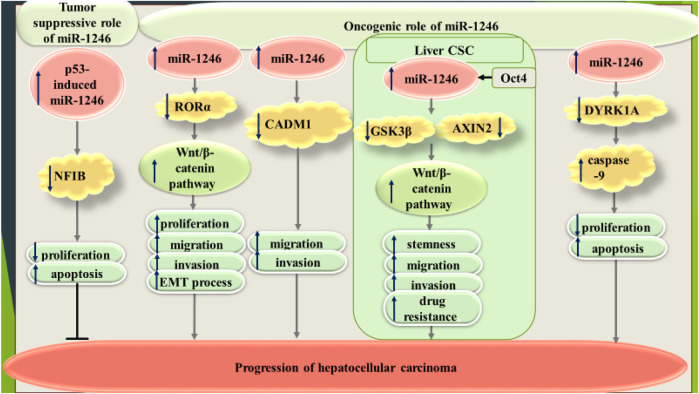
Dual roles of miR-1246 in hepatocellular carcinoma.

On the other hand, Zhang et al. have shown that expression of miR-1246 is induced by p53. This miRNA has been shown to inhibit proliferation of hepatocellular carcinoma cells through influencing expression of NFIB (Zhang et al., 2015). Figure 4 shows dual roles of miR-1246 in hepatocellular carcinoma.

**FIGURE 5:**
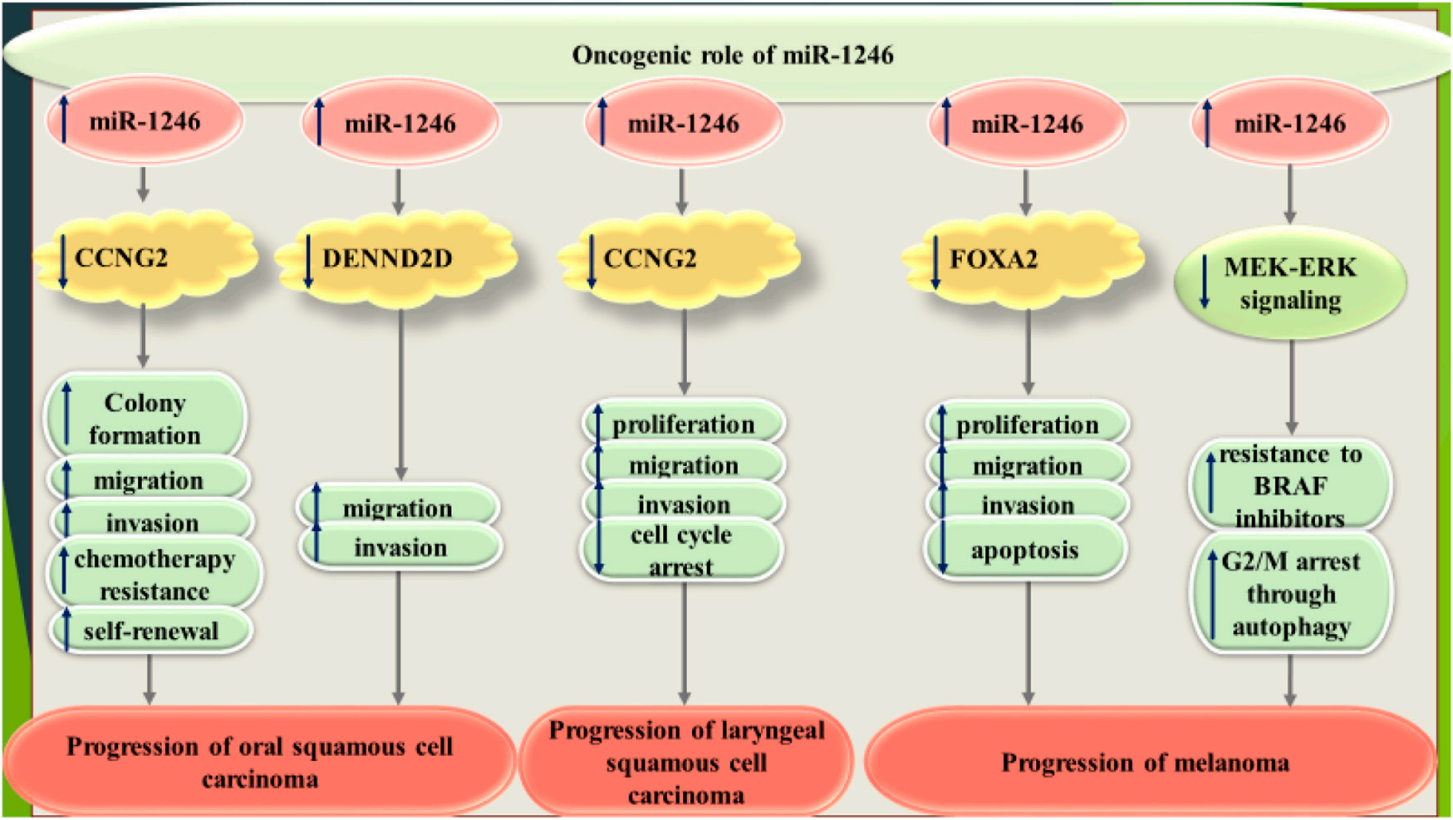
Oncogenic role of miR-1246 in oral and laryngeal squamous cell carcinomas and melanoma.

In oral squamous cell carcinoma, miR-1246 has been shown to target CCNG2 to facilitate stemness properties and induce resistance to chemotherapy (Lin et al., 2018). Moreover, exosomal transfer of this miRNA has enhanced cell motility and invasiveness of oral squamous cell carcinoma cells through targeting DENND2D (Sakha et al., 2016). Consistent with this finding, small extracellular vesicles originated from laryngeal squamous cell carcinoma cells have been shown to enter into neighboring cells. Lack of miR-1246 in these vesicles abolished development of this kind of cancer. miR-1246 content of small vesicles could participate in the pathoetiology of laryngeal squamous cell carcinoma through suppressing CCNG2 expression (Huang Q. et al., 2020). miR-1246 is involved in the progression of melanoma via changing expression levels FOXA2 (Yu et al., 2020). Moreover, miR-1246 has been shown to increase resistance of melanoma cells to BRAF inhibitors (Kim et al., 2017). Figure 5 shows oncogenic role of miR-1246 in oral and laryngeal squamous cell carcinomas and melanoma.

Exosomes originated from glioma cell cultures under hypoxic conditions could shuttle miR-1246 to normoxic glioma cells and enhance their migratory potential and invasiveness (Qian M. et al., 2021). Another study has shown the impact of these exosomes in induction of polarization of macrophages into M2 phenotype through targeting TERF2IP and subsequent influence on the activities of STAT3 and NF-κB signaling (Qian et al., 2020).

In pancreatic cancer, miR-1246 could increase chemoresistance and stemness through modulation of CCNG2 (Hasegawa et al., 2014).

Finally, in ovarian cancer, miR-1246 can confer resistance to chemotherapeutics through influencing Cav1/p-gp/M2-type macrophages (Kanlikilicer et al., 2018).

**FIGURE 6:**
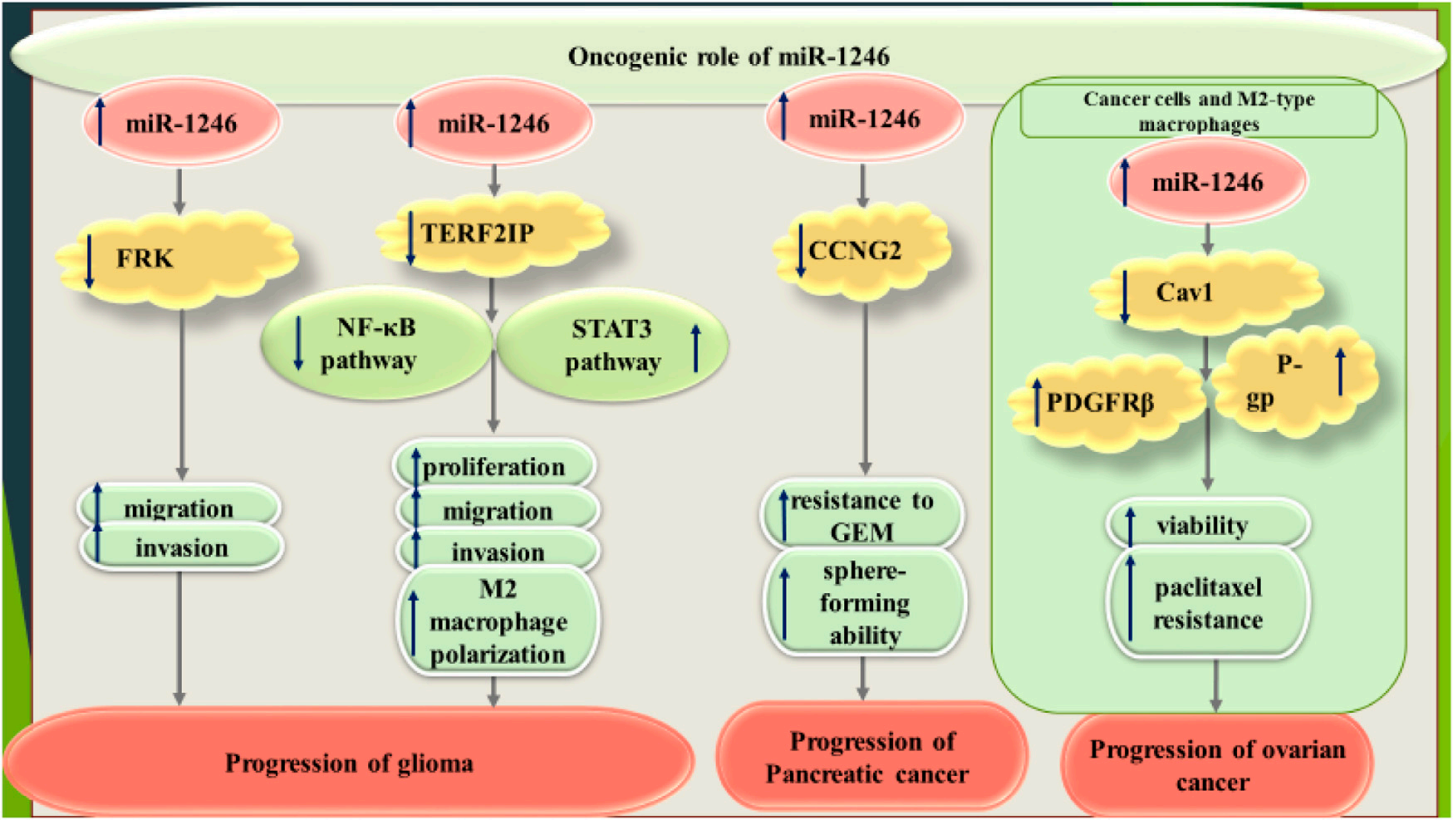
Oncogenic role of miR-1246 in glioma, pancreatic cancer and ovarian cancer.

Figure 6 shows the oncogenic role of miR-1246 in glioma, pancreatic cancer and ovarian cancer.

Table 1 shows the outlines of *in vitro* studies focusing on the function of miR-1246 in cancer.

**TABLE 1:** Outlines of *in vitro* studies about function of miR-1246 (∆: knock-down or deletion, FN: Fusobacterium nucleatum, sEV: Small extracellular vesicle, GEM: gemcitabine).

Tumor type	Targets/Regulators and signaling pathways	Cell line	Function	References
Colorectal cancer	m6A, METTL3, SPRED2, RAF/MEK/ERK pathway	LoVo, HCT116, CaCo2, DLD-1, HT-29, NCM460	∆ METTL3: ↓ migration, ↓ invasion	Peng et al. (2019)
			↑ METTL3: ↑ migration, ↑ invasion	
	GSK3β, Wnt/β-catenin pathway	HCT116, SW480	Fn infection: ↑ secretion of exosomes	Guo et al. (2021)
			Fn-Ex treatment: ↑ migration, ↑ wound closure	
			↑ miR-1246: ↑ migration, ↑ wound closure	
	CycG2	SW620, SW480, HCT116, HT29, LOVO, IECs	∆ miR-1246: ↓ proliferation, ↓ migration, ↓ invasion, ↑ apoptosis	Wang et al. (2016)
			↑ miR-1246: ↑ proliferation, ↑ migration, ↑ invasion, ↓ apoptosis	
	—	HT-29	∆ miR-1246: ↓ proliferation	Nagai et al. (2021)
			↑ miR-1246: ↑ proliferation	
Lung cancer	GSK-3β, Wnt/β-catenin signaling pathway	A549	∆ miR-1246: ↓ migration, ↓ invasion, ↓ EMT process	Yang et al. (2019)
			↑ miR-1246: ↑ migration, ↑ invasion, ↑ EMT process	
	DR5	A549, SK-MES-1, H446	∆ miR-1246: ↓ proliferation, ↑ radiation sensitivity	Yuan et al. (2016)
			↑ miR-1246: ↑ proliferation, ↓ radiation sensitivity	
	—	A549, HCC1588	∆ miR-1246: ↓ proliferation, ↓ stemness, ↓ EMT process, ↓ sphere-formation, ↓ colony formation, ↓ invasion	Kim et al. (2016)
	CXCR4, JAK/STAT and PI3K/AKT signal pathways	A549, H1650, H1299, 16HBE14o	↑ miR-1246: ↓ invasion, ↓ EMT process	Xu et al. (2018)
	METTL3, m6A, PEG3	A549, H1299, H520, H1975	∆ METTL3: ↓viability, ↓ colony formation, ↓ migration, ↓ invasion, ↑ apoptosis	Huang et al. (2021)
			↑ miR-1246: ↑ migration, ↑ invasion, ↓ apoptosis	
Cervical cancer	HPV16E6, DYRK1A	HeLa, SiHa, Caski, C33A	∆ miR-1246:↑ invasion↑ miR-1246: ↓ invasion	Yang et al. (2015)
	THBS2	SiHa	↑ miR-1246: ↑ proliferation, ↑ migration, ↑ invasion	Chen et al. (2014)
			∆ miR-1246: ↓ proliferation, ↓ migration, ↓ invasion	
	THBS2, THBS2/MMP signaling pathway	SiHa	∆ miR-1246: ↓ proliferation, ↑ apoptosis, ↑ G1 phase arrest	Du et al. (2019)
	—	Siha, Hela	radiation treatment: ↑ miR-1246	Zhang et al. (2013)
			↑ miR-1246: ↓ radiosensitivity	
Prostate cancer	—	RWPE-1, LNCaP, Du145, PC3	↑ miR-1246: ↓ proliferation, ↓ migration, ↓ invasion, ↓ EMT process, ↑ apoptosis	Bhagirath et al. (2018)
Breast cancer	CCNG2	MCF-7, MDA-MB-231, MCF-10A, HMLE	↑ miR-1246: ↑ proliferation, ↑ migration, ↑ invasion, ↑ chemotherapy resistance	Li et al. (2017)
Hepatocellular carcinoma	RORα, Wnt/β-catenin pathway	HSCs, PLC, MHCC97H, HCCLM3	↑ miR-1246: ↑ proliferation, ↑ migration, ↑ invasion, ↑ EMT process	Huang et al. (2020a)
	CADM1	HepG2, SMMC7721 and BEL7402	∆ miR-1246: ↓ migration, ↓ invasion	Sun et al. (2014)
			↑ miR-1246: ↑ migration, ↑ invasion	
	p53, NFIB	HepG2, Hep3B, Huh7, C3A, PLC, LO2, SUN387	∆ miR-1246: ↓	Zhang et al. (2015)
			Proliferation	
			↑ miR-1246: ↑ proliferation	
	Oct4, AXIN2, GSK3β, Wnt/β-catenin pathway	Hep3B, Huh7	∆ miR-1246: ↓ invasion, ↓ migration, ↓ ability to initiate hepatosphere formation, ↓ self-renewal, ↑ sensitization to 5-fluorouracil, cisplatin and sorafenib	Chai et al. (2016)
	Galectin-9, DYRK1A, caspase-9	Li-7, Huh7, HLE	↑ galectin-9: ↓ Proliferation, ↑ apoptosis, ↑ miR-1246	Fujita et al. (2015)
			↑ miR-1246+ galectin-9 treatment in Li-7 cells: ↓ Proliferation, ↑ apoptosis	
	—	BEL-7402	bafilomycin A1-treatment: ↓	Lu et al. (2015)
			Proliferation, ↓ invasion, ↑ miR-1246	
Oral squamous cell carcinoma	CCNG2	OC3, FaDu	∆ miR-1246: ↓ migration, ↓ invasion, ↓ self-renewal, ↓ colony formation, ↓ chemoresistance	Lin et al. (2018)
			↑ miR-1246: ↑ invasion, ↑ colony formation, ↑ number of spheres, ↑ stemness	
	DENND2D	HOC313-P, TSU, HeLa	↑ miR-1246: ↑ migration, ↑ invasion, did not affect growth	Sakha et al. (2016)
Laryngeal squamous Cell carcinoma	CCNG2	Hep-2, AMC-HN-8	∆ miR-1246 in sEV: ↓	Huang et al. (2020b)
			Proliferation, ↓ migration, ↓ invasion, ↑ cell cycle arrest	
Melanoma	FOXA2	HEM, A375, A2058	∆ miR-1246 in sEV: ↓	Yu et al. (2020)
			Proliferation, ↓ migration, ↓ invasion, ↑ apoptosis	
			↑ miR-1246: ↑ proliferation, ↑ migration, ↑ invasion, ↓ apoptosis	
	MEK-ERK signaling	A375P BRAF V600E, A375P/Mdr, SK-MEL-2 BRAF-WT	↑ miR-1246: ↑ resistance to BRAF inhibitors, ↑ G2/M arrest through autophagy	Kim et al. (2017)
	IL-6, STAT3, Akt	HMVECs, A375, A375SM	↑ miR-1246: ↑ resistance to 5-FU	Torii et al. (2021)
Glioma	FRK	H-GDEs	↑ miR-1246: ↑ migration, ↑ invasion	Qian et al. (2021a)
	TERF2IP, STAT3 pathway, NF-κB pathway	U87MG, U251, U937	↑ miR-1246: ↑ proliferation, ↑ migration, ↑ invasion, ↑ M2 macrophage polarization	Qian et al. (2020)
Pancreatic cancer	CCNG2	Panc1, Panc1-P	↑ miR-1246: ↑ resistance to GEM, ↑ sphere-forming ability	Hasegawa et al. (2014)
Ovarian cancer	Cav1, PDGFRβ, P-gp	eyA8, SKOV3-ip1, A2780, HeyA8-MDR, SKOV3-TR, A2780-CP20, HIO180	∆ miR-1246: ↓ paclitaxel resistance, ↓ viability	Kanlikilicer et al. (2018)
	—	HO-8910	bafilomycin A1-treatment: ↓	Lu et al. (2015)
			Proliferation, ↓ invasion, ↑ miR-1246	
Clear cell renal cell carcinoma	GABPB1-AS1, PCK1	786-o and caki-1	↑ GABPB1-AS1: ↓	Gao et al. (2020)
			Proliferation, ↓ migration, ↓ invasion	
Acute myeloid leukemia	LRIG1, STAT3 pathway	LSCs	↑ miR-1246 + LSCs co-cultured with EVs: ↑ viability, ↑ colony formation, ↓ apoptosis, ↓ differentiation	Chen et al. (2021a)
	Raptor/mTOR pathway	Molm-14, HL-60, U-937, LT-HSC	↑ miR-1246: ↓ protein synthesis, ↑ quiescence	Abdelhamed et al. (2019)
Leukemia	AXIN2, GSK-3β, Wnt/β-catenin pathway, P-gp	K562, HL-60 cells and drug-resistant K562/ADM, HL-60/RS	∆ miR-1246: ↓ Proliferation, ↑ apoptosis, ↑ chemo-sensitivity	Xie et al. (2021)
T cell acute lymphoblastic leukemia (T-ALL)	NOTCH2 Pathway	T-ALL	∆ miR-1246: ↓ Proliferation	Luo et al. (2018)
			↑ miR-1246: ↑ Proliferation	
Gastric cancer	Oxidative stress response, axon guidance mediated by netrin, salvage pyrimidine deoxyribonucleotides	NCI-N87	cisplatin treatment: ↑ miR-1246	Yin et al. (2019)
Gallbladder cancer	—	G415	∆ miR-1246: ↓ Proliferation, ↓ invasion, ↑ apoptosis	Ueta et al. (2021)
			↑ miR-1246: ↑ Proliferation, ↑ invasion, ↓ apoptosis	
Sarcoma	—	LP6, LPS12	↑ miR-1246: ↑ Proliferation	Kohama et al. (2021)

## Animal Studies

Most of animal studies have indicated an oncogenic role for miR-1246, since its silencing has led to reduction of tumor size and attenuation of tumor growth (Table 2). Moreover, expression of miR-1246 has been found to be elevated in the plasma exosomes of patient-originated orthotopic xenograft animals compared to control animals (Hannafon et al., 2016). However, in prostate cancer, miR-1246 up-regulation has significantly inhibited tumor growth in the xenograft models, suggesting its tumor suppressive role. Moreover, in miR-1246 overexpressing xenograft models, exosomal levels of this miRNA has been reduced. Taken together, miR-1246 has been identified as a tumor suppressor miRNA being selectively packaged in prostate cancer exosomes, resulting in its high abundance in serum yet low concentrations inside the cells (Bhagirath et al., 2018). In the xenograft model of leukemia, miR-1246-containing extracellular vesicles have been shown to confer quiescence on residual hematopoietic stem cells (Abdelhamed et al., 2019).

**TABLE 2:** Outline of studies about the function of miR-1246 in animal models (∆: knock-down or deletion, PDX: derived orthotopic xenograft, NOD-SCID: non-obese diabetic/severe combined immunodeficiency, NSG: NOD Cg-Prkdcscid Il2rgtm1Wjl/SzJ).

Tumor type	Animal models	Results	References
Colorectal cancer	Male BALB/c nude mice	∆ METTL3: ↓ metastasis	Peng et al. (2019)
	BALB/c nude mice	∆ miR-1246 in Fn-Ex group: ↓ metastasis	Guo et al. (2021)
Lung cancer	Female BALB/c nude mice	∆ METTL3: ↓ tumor volume, ↓ tumor weight	Huang et al. (2021)
		↑ miR-1246: ↑ tumor volume, ↑ tumor weight	
Cervical cancer	Athymic BALB/c nude mice	∆ miR-1246: ↓ tumor volume, ↓ tumor growth	Du et al. (2019)
Prostate cancer	Nude mice	↑ miR-1246 in xenograft tissues: ↓ tumor growth	Bhagirath et al. (2018)
Breast cancer	Plasma of a PDX mouse	miR-1246 was higher in the plasma exosomes of patient-PDX mice compared to control mice	Hannafon et al. (2016)
Hepatocellular carcinoma	Male BALB/c-nu/nu mice	↑ miR-1246: ↑ tumor growth, ↑ metastasis	Huang et al. (2020a)
	Male BALB/c nude or NOD-SCID mice	∆ miR-1246: ↓ tumor initiation, ↓ tumor volume, ↓ metastasis	Chai et al. (2016)
	Female athymic BALB/c-nu/nu mice	↑ galectin-9: ↓ tumor growth of Li-7 cells, ↑ apoptosis, ↑ miR-1246	Fujita et al. (2015)
Oral squamous cell carcinoma	BALB/c nude mice	∆ miR-1246: ↓ tumor size	Lin et al. (2018)
		↑ miR-1246: ↑ tumor growth	
Glioma	Male nude mice	↑ miR-1246: ↑ proliferation, ↑ M2 macrophage polarization	Qian et al. (2020)
Pancreatic cancer	Female non-obese mice with diabetes/severe combined immunodeficiency	↑ miR-1246 in Panc1-P-l-OE: ↑ tumourigenicity	Hasegawa et al. (2014)
Ovarian cancer	Nude mice	∆ miR-1246 + chemotherapy: ↓ tumor weight, ↓ macrophages recruited by tumors	Kanlikilicer et al. (2018)
Acute myeloid leukemia (AML)	NOD/SCID mice	↓ EVs-miR-1246: ↓ tumor volume, ↓ tumor weight	Chen et al. (2021a)
	NSG and C57BL/6J mice	↑ miR-1246: ↑ quiescence	Abdelhamed et al. (2019)
Leukemia	Male BALB/c nude mice	∆ miR-1246: ↓ tumor volume, ↓ tumor weight, ↓ chemotherapy resistance	Xie et al. (2021)

## Clinical Studies

Serum levels of miR-1246 have been found to be higher in the sera of colorectal cancer patients compared to healthy subjects (Salah et al., 2020). Similarly, miR-1246 has been found as the most up-regulated miRNA in the sera of patients with lung cancer (Yang et al., 2019). Levels of miR-1246 have been found to be higher in laryngeal squamous cell carcinoma tissues and plasma small extracellular vesicles. This miRNA has been more enriched in small extracellular vesicles instead of being in soluble form (Sakha et al., 2016). Almost all studies in clinical settings have reported up-regulation of miR-1246 in neoplastic tissues and sera of patients compared with controls (Table 3).

**TABLE 3:** Results of studies that reported dysregulation of miR-1246 or other genes that interact with miR-1246 in clinical samples.

Tumor type	Samples	Expression of miR-1246 or other genes (tumor vs. normal)	Kaplan-Meier analysis (impact of miR-1246 dysregulation or other genes dysregulation)	Univariate/Multivariate cox regression	Association of expression of miR-1246 or expression of other genes with clinicopathologic characteristics	Method for assessment of miR-1246 expression	References
Colorectal cancer (CRC)	60 pairs of CRC tissues and ANCTs	Up-regulation of m6A	—	—	lymph node invasion, and distant metastasis	SYBR Premix Ex Taq Kit	Peng et al. (2019)
		Up-regulation of METTL3					
	GEO database: GSE17536	Up-regulation of METTL3	Lower OS	—	—		
	Serum samples from 82 patients and blood samples from 102 healthy controls	Up-regulation of miR-1246	—	—	—	Mir-X miRNA RT-qPCR TB Green Kit	Guo et al. (2021)
	40 CRC Patients and 40 healthy controls	Up-regulation of exosomal miR-1246	—	—	—		
	82 fecal samples	Up-regulation of miR-1246	—	—	Abundance of Fn		
	sera of 37 CRC patients and 30 healthy controls	Up-regulation of miR-1246	—	—	—	miScript syber green PCR kit (Qiagen)	Salah et al. (2020)
	10 pairs of CRC tissues and ANCTs	Up-regulation of miR-1246	—	—	—	mirVana™ qRT-PCR microRNA detection kit	Wang et al. (2016)
	Serum samples from 43 CRC patients	Up-regulation of miR-1246 in chemoresistant patients	—	—	—	TaqMan miRNA Assay	Jin et al. (2019)
	26 CRC patients	Up-regulation of miR-1246 (lower in post-treatment sera)	—	—	—	Taqman miRNA Assay	Handa et al. (2021)
	181 pairs of CRC tissues and ANCTs	Up-regulation of miR-1246	—	—	CD44v6 status	SYBR Green	Toden et al. (2019)
	150 pairs of CRC tissues and ANCTs	Up-regulation of miR-1246	worse OS and DFS	miR-1246 was found to be an independent prognostic factor for OS and DFS	stage IV		
Colorectal cancer (CRC) and Colorectal adenomas (CRA)	6 CRC and 8 CRA patients	Up-regulation of miR-1246 in both cellular compartments and exosomes (higher in CRC-derived organoids than CRA-derived organoids)	—	—	—	TaqMan^TM^Advanced miRNA assays	Nagai et al. (2021)
Colon cancer	88 primary CRC patients and 11 healthy controls	Up-regulation of miR-1246	—	—	—	TaqMan microRNA kits	Ogata-Kawata et al. (2014)
	Serum from 29 of the patients after surgical resection	Down-regulation of miR-1246	—	—	—		
	13 CRC patients and 8 healthy controls	Up-regulation of miR-1246	—	—	—		
Lung cancer	serum from 11 lung cancer patients and 5 healthy control	Up-regulation of miR-1246	—	—	metastasis	SYBR Green	Yang et al. (2019)
	105 NSCLC patients, 50 NMRD patients, and 50 healthy controls	Up-regulation of miR-1246 in NSCLC patients than in patients with NMRD and healthy controls	Worse OS and DFS	Serum exosomal miR-1246, TNM stage, and lymph node metastasis were found as independent prognostic factors for OS.	advanced clinical stage and with lymph node metastasis	miScript SYBR-Green PCR Kit	Huang and Qu (2020)
	GEO database: GSE137140 and GSE69732	Up-regulation of miR-1246	High expression of UBE2C, UCHL1, TRAIP, TNNT1, TNNI3, RAC3 (PTGs of cmiRNA-1246) = poor OS	—	—	—	Huang et al. (2020c)
			High expressions of PITX2, NRAS, ENFA4, DNAJA3, TBCE, and TGIF1(PTGs of cmiRNA-1246) = longer OS in LUAD patients				
Lung cancer	86 pairs of NSCLC tissues and ANCTs	Up-regulation of METTL3 (that upregulates miR-1246)	—	—	Lymph node metastasis, tumor size, and TNM stage	TaqMan MicroRNA Assays	Huang et al. (2021)
	86 pairs of NSCLC tissues and ANCTs	Up-regulation of m6A and Up-regulation of miR-1246	—	—	—		
	52 NSCLC patients and 45 healthy controls	Up-regulation of miR-1246	—	—	—	QIAGEN SYBR green Master Mix	Zheng et al. (2021)
Cervical cancer	68 cervical cancer patients and 52 healthy controls	Down-regulation of miR-1246	—	—	Advanced clinical stage, invasive cervical wall N1/2, HPV positivity	Stem-loop primers using SYBR® Premix Ex Taq™ II kit	Yang et al. (2015)
	18 pairs of cervical cancer tissues and ANCTs	Down-regulation of miR-1246	—	—	—		
	26 cervical cancer patients and 16 healthy controls	Up-regulation of miR-1246	—	—	—	TaqMan miRNA RT-Kit with stem-loop RT-primer	Nagamitsu et al. (2016)
Esophageal cancer	Serum from 55 ESCC patients and 39 healthy controls	Up-regulation of miR-1246	—	—	—	miScript SYBR®-Green PCR Kit (Qiagen)	Hoshino et al. (2020a)
	Serum from 101 ESCC patients and 34 healthy controls	Up-regulation of miR-1246	Worse 5-years OS and DFS	_	Tumor depth, positive lymph node metastasis, stage, and survival of patients		
	101 ESCC patients and 35 healthy controls	Up-regulation of miR-1246	—	miR-1246real was found to be an independent factor for N stage and miR-1246real, and miR-1246pred was found to be an independent factor for N stage and miR-1246pred	—	miScript SYBR®-Green PCR Kit (Qiagen	Hoshino et al. (2020b)
	Serum from 101 ESCC patients and 46 healthy controls	Up-regulation of miR-1246	lower 2-years OS	Expression of miR-1246 was the strongest independent risk factor for a poor survival with a hazard ratio of 4.032	T3–4, lymph node metastasis, distant metastasis, stage III–IV	TaqMan MicroRNA Assays	Takeshita et al. (2013)
	22 pairs of ESCC tissues and ANCTs	No significant differences	—	—	—		
	32 lymph nodes	Higher in proximal lymph nodes than abdominal lymph nodes, thoracic lymph nodes, and cervical lymph nodes	—	—	—		
	Serum from 72 ESCC patients and 50 healthy controls	Up-regulation of miR-1246	worse OS	—	Tumor invasion and positive lymph node metastasis, albeit insignificantly	miScript SYBR® Green PCR kit (Qiagen)	Hoshino et al. (2021)
	Urine from 72 ESCC patients and 50 healthy controls	Up-regulation of miR-1246	—	—	—	—	
Prostate cancer (PCa)	Serum from 6 PCa patients, 3 BPH patients and 3 healthy controls	Up-regulation of miR-1246 in PCa than BPH and healthy controls	—	—	Advancing PCa stage, lymph node metastasis	TaqMan MicroRNA Assays	Bhagirath et al. (2018)
	Serum form 44 PCa patients, 4 BPH patients and 8 healthy controls	Up-regulation of ex-miR-1246 in PCa than BPH and healthy controls	—	—	Advancing PCa stage, lymph node metastasis		
	36 pairs of PCa tissues and ANCTs	Down-regulation of miR-1246	—	—	—		
Breast cancer	Circulating exosomal miRNA from 4 trastuzumab-resistant and 4 sensitive patients	Up-regulation of miR-1246 in trastuzumab-resistant HER2-positive breast cancer patients	poorer EFS	Expression of miR-1246 strongly showed poor EFS for early-stage patients, and poor PFS for metastatic patients	—	miScript SYBR Green PCR Kit (Qiagen)	Zhang et al. (2020)
	Plasma from 16 breast cancer patients and 16 healthy controls	Up-regulation of miR-1246 in plasma exosomes	—	—	—	Stem-loop primer using TaqMan microRNA Reverse Transcription Kit	Hannafon et al. (2016)
	Serum from 56 breast cancer patients and 19 healthy controls	Up-regulation of miR-1246	—	—	—	Taqman assay	Li et al. (2017)
	11 studies with 921 breast cancer patients	Up-regulation of miR-1246	—	—	—	—	Wang et al. (2018a)
Breast cancer	Serum from 100 breast cancer patients and 40 healthy controls	Up-regulation of miR-1246	—	—	—	miScript SYBR Green PCR kit (Qiagen GmbH)	Fu et al. (2016)
	GEO database: (GSE73002) (1,288 BC patients and 2,686 healthy controls)	Up-regulation of miR-1246	—	—	—	—	Cui et al. (2018)
	GEO database: (GSE73002) (429 BC patients and 895 healthy controls.)	Up-regulation of miR-1246	—	—	—		
Hepatocellular carcinoma (HCC)	Serum from 33 primary HCC patients, 22 metastatic liver tumor patients, 30 healthy controls	Up-regulation of miR-1246 in metastatic liver tumors	—	—	Females, patients ≤60 years old, and patients with cirrhosis and low level of serum AFP	miScript SYBER Green PCR kit (Qiagen)	Ahmed et al. (2019)
	7 HCC patients, 21 cirrhosis patients and 14 healthy controls	Up-regulation of miR-1246	—	—	—	QX200 EvaGreen ddPCR protocol	Moshiri et al. (2018)
	Plasmas from 9 HCC and 6 cirrhotic patients	Up-regulation of miR-1246	—	—	—		
	Plasmas from 22 HCC patients and 11 healthy controls	Up-regulation of miR-1246	—	—	—		
	Plasmas from 24 HCC and 14 cirrhotic patients	Up-regulation of miR-1246	—	—	—		
	Serum from 50 HCC patients and 50 healthy controls	Up-regulation of miR-1246	shorter OS	—	—	miScript SYBR-Green PCR Kit (Qiagen GmbH)	Chen et al. (2021b)
	50 pairs of HCC tissues and ANCTs	Up-regulation of miR-1246	—	—	TNM staging, differentiation, and metastasis		
	31 pairs of HCC tissues and ANCTs	Up-regulation of miR-1246	—	—	—	An Agilent oligonucleotide microarray system (Agilent Gene Spring GX11.51, Agilent Technologies)	Huang et al. (2020a)
	Serum from 121 HCC patients, 48 CH patients, 25 LC patients and 15 healthy controls	Up-regulation of miR-1246 in HCC compared to CH, LC, HC	shorter OS and DFS	Serum miR-1246, Albumin, AFP-L3, tumor differentiation, and were independently correlated with poor prognosis	UICC-TNM classification, tumor differentiation, and pathological portal vein invasion	TaqMan Advanced miRNA Assays	Chuma et al. (2019)
	38 liver cancer patients	Up-regulation of miR-1246	shorter DFS	—	—	miScript SYBR Green PCR kit (Qiagen)	Sun et al. (2014)
	28 pairs of HCC tissues and ANCTs	Expression of miR-1246 was consistent with p53 levels	—	—	—	SYBR PremixEx Taq™	Zhang et al. (2015)
	62 HCC patients received liver transplantation	Up-regulation of miR-1246 in HCC recipients with HCC recurrence after liver transplantation than those without tumor recurrence	Poor OS and DFS after liver transplantation	Early-phase circulating miR-1246 was found to be significant predictor for predicting OS and DFS of HCC recipients	The serum AST level from day 0 to day 3, serum ALT level from day 0 to day 6 after liver transplantation, and expression of TNF-a	TaqMan MicroRNA Assays	Ng et al. (2016)
	114 pairs of HCC tissues and ANCTs	Up-regulation of miR-1246	Worse OS and DFS	miR-1246 was an independent prognostic factor for both OS and DFS.	Serum alpha fetoprotein (AFP) level	—	Chai et al. (2016)
	5 LC and 5 HCC patients	Higher in HCC than in LC	—	—	—	TaqMan microRNA Reverse Transcription Kit	Wang et al. (2018b)
	10 CH, 13 LC, 18 HCC patients and 14 healthy controls	Higher in HCC than in LC and CH groups, not different from NC group	—	—	—		
	40 CH, 40 LC, 50 HCC patients and 50 healthy controls	Up-regulation of miR-1246 in HCC than in LC and NC groups	—	—	—		
Oral squamous cell carcinoma (OSCC)	30 pairs of OSCC tissues and ANCTs	Up-regulation of miR-1246	poor OS	—	T category, stage, and lymph node metastasis	TaqMan miRNA assays	Lin et al. (2018)
	106 pairs of OSCC tissues and ANCTs	Up-regulation of miR-1246	poor OS	miR-1246 expression, tumor grade and TNM stage were independent prognostic factors for OSCC.	TNM stage, nodal status, and tumor grade	SYBR PrimeScript miRNA RT-PCR kit	Liao et al. (2015)
	Plasma from 10 advanced OSCC patients and 10 healthy controls	Up-regulation of miR-1246	—	—	—	miScript SYBR Green PCR kit (QIAGEN)	Nakashima et al. (2019)
	55 advanced OSCC patients	Up-regulation of miR-1246	—	—	Tumor stage		
Laryngeal squamous cell carcinoma (LSCC)	Plasma from 61 LSCC patients, 26 healthy controls	Up-regulation of miR-1246	poor OS	—	—	miScript SYBR Green PCR Kit (QIAGEN)	Huang et al. (2020b)
	61 pairs of LSCC tissues and ANCTs	Up-regulation of miR-1246	poor OS	—	—		
	GEO database: (GSE124678, GSE70289, GSE62819) (14 LSCC tissues and 49 ANCTs)	Up-regulation of miR-1246	—	—	—	—	Jing et al. (2020)
Pancreatobiliary tract cancer	12 pancreatobiliary tract cancer patients and 13 healthy controls	Up-regulation of miR-1246	—	—	—	TaqMan MicroRNA Assays	Machida et al. (2016)
Melanoma	43 pairs of melanoma tissues and ANCTs	Up-regulation of miR-1246	—	—	—	SYBR-Green Premix Ex Taq II	Yu et al. (2020)
	42 melanoma patients and 20 healthy controls	Up-regulation of miR-1246 in melanoma patients’ EVs	—	—	metastatic tumor EVs	KAPA SYBR Fast qPCR Kit	Torii et al. (2021)
Glioma	26 glioma patients	Up-regulation of miR-1246 in GBM than LGG patients	—	—	—	SYBR Premix Ex Taq™ Kit	Qian et al. (2020)
	CGGA, GEO Databases: (GSE25632, GSE104554) (311 glioma patients)	Up-regulation of miR-1246	Worse prognosis	miR-1246 was an independent risk factor for OS.	Tumor recurrence	—	Ji et al. (2020)
Pancreatic cancer (PC)	Plasma from 15 PC patients and 15 healthy controls	Up-regulation of miR-1246	—	—	—	qScript miRNA cDNA Synthesis Kit	Xu et al. (2017)
	7 PDAC patients, 4 IPMN patients, 4 NET patients	Up-regulation of miR-1246 in patients with IPMN	—	—	—		
	GEO datasets (GSE113486, GSE106817, GSE59856)	Up-regulation of miR-1246	—	—	—	Hairpin-itTM microRNA RT-PCR Quantitation Kit	Wei et al. (2020)
	120 PC patients, 40 benign pancreatic disease controls (DC) and 40 healthy controls	Up-regulation of miR-1246 in PC than DC and HC group	—	miR-1246 was significant and independent risk factors for PC​	Tumor size		
	Serum from 34 pairs of pre- and post-operation PC patients	Down-regulation of miR-1246 after surgical resection of malignancies	—	—	—		
Ovarian cancer (OC)	15 OC tissues and 7 normal ovarian surface epithelium tissues	Up-regulation of miR-1246 in OC exosomes	—	—	Paclitaxel-resistant	PerfeCTa microRNA Assay Kit	Kanlikilicer et al. (2018)
	Serum from 110 HGSOC patients and 52 Healthy controls	Up-regulation of miR-1246	—	—	—	Rotor-Gene Thermal Cycler (Qiagen	Todeschini et al. (2017)
	serum from 58 HGSOC patients and 13 Healthy controls	Up-regulation of miR-1246	—	—	—		
	59 high-grade OSC patients	Up-regulation of miR-1246	—	—	—	TaqMan microRNA reverse transcription kit	Cha et al. (2017)
Clear cell renal cell carcinoma (ccRCC)	48 pairs of ccRCC tissues and ANCTs	Down-regulation of GABPB1-AS1 (that sponges miR-1246)	better OS	—	inversely associated with tumor size, TNM stage, and Furhman stage	Roche PCR system	Gao et al. (2020)
Gastric cancer (GC)	urine from and 7 GC patients and 3 healthy controls	Up-regulation of miR-1246	—	—	—	Illumina NextSeq 500 SE50 (20M) sequencing	Qian et al. (2021b)
Gallbladder cancer (GBC)	Serum EVs from 3 patients with GBC, 3 with Benign and 10 healthy controls	Up-regulation of miR-1246 in GBC than Benign and healthy controls	—	Serum EV miR-1246 was significant independent prognostic factor	Advanced-stage GBC	—	Ueta et al. (2021)
	GEO database: GSE104165, GSE112408	Up-regulation of miR-1246 in GBC tissues	—	—	—		
Sarcoma	22 Sarcoma patients, 17 DDLPS patients, and 3 EWS patients	Up-regulation of miR-1246 in DDLPS	—	—	—	miScript®SYBR®Green PCR kit	Kohama et al. (2021)

However, Yang et al. have shown down-regulation of miR-1246 in cervical cancer tissues compared with normal controls. Notably, down-regulation of miR-1246 has been inversely correlated with clinical stage and HPV16 E6 infection. Yet, its levels have not been correlated with age, tumor diameters, invasion deepness, lymph node involvement, or vascular invasion (Yang et al., 2015).

Table 3 Results of studies that reported dysregulation of miR-1246 or other genes that interact with miR-1246 in clinical samples (OS: Overall survival, DFS: disease-free survival, TNM: tumor-node-metastasis, ANCTs: adjacent non-cancerous tissues, FN: Fusobacterium nucleatum, CD44v6: a CSC population with increased resistance to chemotherapeutic agents, NMRD: non-malignant respiratory diseases, NSCLC: non-small cell lung cancer, PTGs: potential target genes, LUAD: lung adenocarcinoma, ESCC: esophageal squamous cell carcinoma, miR-1246real and miR-1246pred: real and predicted miR-1246 expression levels, BPH: benign prostate hyperplasia, EFS: event-free survival, PFS: progression-free survival, LC: liver cirrhosis, CH: chronic hepatitis, HC: healthy controls, UICC: Union for International Cancer Control, GBM: glioblastoma, LGG: low-grade glioma, PDAC: pancreatic ductal adenocarcinomas, IPMN: intraductal papillary mucinous neoplasms, NET: well differentiated neuroendocrine tumors, HGSOC: High-grade serous ovarian carcinoma, OSC: ovarian serous carcinoma, EVs: extracellular vesicles, DDLPS: dedifferentiated liposarcoma, EWS: Ewing’s sarcoma).

Diagnostic value of miR-1246 has been validated in different neoplastic disorders (Table 4). The most promising results have been revealed in breast cancer. This miRNA could separate breast cancer patients from healthy controls with area under receiver operating characteristic curve (AUC) of 0.967 (Cui et al., 2018). In hepatocellular carcinoma, miR-1246 could be used as a diagnostic marker for differentiation of cancer status from cirrhosis and healthy controls with AUC values of 0.97 and 0.83, respectively (Moshiri et al., 2018). Expression level of miR-1246 in serum samples have been shown to distinguish colorectal cancer patients from healthy subjects with sensitivity of 100% and specificity of 80% (Salah et al., 2020). This miRNA could separate lung cancer patients from healthy controls with AUC value of 0.82 (Huang and Qu, 2020). Moreover, serum and urine levels of miR-1246 could be used as diagnostic markers for esophageal cancer with AUC values of 0.91 and 0.82, respectively (Hoshino et al., 2021).

**TABLE 4:** Diagnostic value of miR-1246 in cancers (NMRD: non-malignant respiratory diseases, NSCLC: non-small cell lung cancer, ESCC: esophageal squamous cell carcinoma, ETR: Early tumor recurrence, HGSOC: High-grade serous ovarian carcinoma).

Tumor type	Numbers of clinical samples	Distinguish between	Area under curve	Sensitivity (%)	Specificity (%)	Accuracy (%)	References
Colorectal cancer (CRC)	Sera of 37 CRC patients and 30 healthy controls	37 CRC patients vs. healthy controls	—	100	80	—	Salah et al. (2020)
	Serum samples from 43 CRC patients	Chemoresistant CRC patients vs. chemosensitive group	0.749	—	—	—	Jin et al. (2019)
Lung cancer	105 NSCLC patients, 50 NMRD patients, and 50 healthy controls	NSCLC patients vs. healthy controls	0.827	—	—	—	Huang and Qu (2020)
		NSCLC patients vs. NMRD patients	0.757	—	—	—	
	52 NSCLC patients and 45 healthy controls	NSCLC patients vs. healthy controls	0.6761	—	—	—	Zheng et al. (2021)
Esophageal cancer	Serum from 55 ESCC patients and 39 healthy controls	ESCC patients vs. healthy controls	0.816	72.7	69.2	—	Hoshino et al. (2020a)
	Serum from 101 ESCC patients and 34 healthy controls	ESCC patients vs. healthy controls	0.779	71.3	70.6	—	
	101 ESCC patients and 35 healthy controls	ESCC patients vs healthy controls	0.754	71.29	73.91	—	Hoshino et al. (2020b)
	101 ESCC patients and 46 healthy controls	ESCC patients vs. healthy controls	0.754	71.3	73.9	—	Takeshita et al. (2013)
	serum 72 ESCC patients and 50 healthy controls	ESCC patients vs. healthy controls	0.912	91.7	76.0	—	Hoshino et al. (2021)
	urine from 72 ESCC patients and 50 healthy controls	ESCC patients vs. healthy controls	0.823	90.3	62.0	—	
Prostate cancer (PCa)	26 lymph node metastatic PCa, 43 non-metastatic PCa, and 8 healthy controls	Non-metastatic vs. localized metastatic PCa patients	0.648	81	∼59	—	Bhagirath et al. (2018)
	43 metastatic castration-resistant PCa cases	Normal and aggressive PCa patients and normal controls	0.933	88.37	100	—	
Breast cancer	32 trastuzumab-resistant patients and 36 trastuzumab sensitive patients	Trastuzumab-resistant patients vs. trastuzumab sensitive patients	0.750	78.1	75	—	Zhang et al. (2020)
	Plasma from 16 breast cancer patients and 16 healthy controls	Breast cancer patients vs. healthy controls	0.69	—	—	—	Hannafon et al. (2016)
	serum from 100 breast cancer patients and 40 healthy controls	Breast cancer patients vs. healthy controls	0.904	93.0	75.0	—	Fu et al. (2016)
	Plasma from 146 breast cancer patients and 90 healthy controls	Breast cancer patients vs. healthy controls	0.95	85.0	93.0	88.0	Jang et al. (2021)
	Plasma from 80 breast cancer patients and 56 healthy controls	Breast cancer patients vs. healthy controls	0.963	86.0	96.0	90.0	
	859 BC patients and 1,791 healthy controls	Breast cancer patients vs. healthy controls	0.967	89.8	91.7	—	Cui et al. (2018)
Hepatocellular carcinoma (HCC)	Serum from 33 primary HCC patients, 22 metastatic liver tumor patients	HCC patients vs. metastatic liver tumor patients	0.708	72.2	67.8	—	Ahmed et al. (2019)
	16 HCC patients and 27 cirrhosis patients	HCC patients vs. cirrhotic patients	0.97	86.7	84.6	85.7	Moshiri et al. (2018)
	29 HCC patients and 25 healthy controls	HCC patients vs. healthy controls	0.83	57.1	78.6	71.4	
	Serum from 50 HCC patients and 50 healthy controls	HCC patients vs. healthy controls	0.865	82.0	80.0	—	Chen et al. (2021b)
	37 HCC patients with ETR and 84 HCC patients without ETR	HCC patients with ETR vs. HCC patients without ETR	0.762	54.1	77.4	—	Chuma et al. (2019)
	62 HCC patients received liver transplantation	High group vs. low group for tumor recurrence	0.775	88.9	66.0	—	Ng et al. (2016)
Pancreatobiliary tract cancer	12 pancreatobiliary tract cancer patients and 13 healthy controls	Pancreatobiliary tract cancer patients vs. healthy controls	0.814	0.667	1.000	—	Machida et al. (2016)
Ovarian cancer (OC)	Serum from 168 HGSOC patients and 65 Healthy controls	HGSOC patients vs. Healthy controls	0.89	87	77	84	Todeschini et al. (2017)

## Discussion

miR-1246 is a miRNA with essential impact on carcinogenic events in different tissues. It exerts oncogenic roles in colorectal, breast, renal, oral, laryngeal, pancreatic and ovarian cancers as well as melanoma and glioma. However, in lung, cervical and liver cancers, studies have reported contradictory results regarding the role of miR-1246. Although several targets have been found for miR-1249 using bioinformatics tools and luciferase assay, CCNG2 is the most appreciated target of this miRNA in the context of cancer. miR-1246/CCNG2 axis not only regulates cell proliferation and cell cycle progression, but also is involved in chemoresistant phenotype.

The main mechanism of dysregulation of miR-1246 in cancer is methylation of pri-miR-1246 by methyltransferase METTL3 and modulation of maturation of pri-miR-1246. Unlike other miRNAs, the role of sponging lncRNAs on its expression is less studied.

miR-1246 has been reported to regulate activity of RAF/MEK/ERK, GSK3β, Wnt/β-catenin, JAK/STAT, PI3K/AKT, THBS2/MMP and NOTCH2 pathways. The role of miR-1246 in response to therapeutic modalities has been verified in different settings, indicating its crucial roles in determination of response to targeted therapies, radiotherapy as well as chemotherapy. In fact, miR-1246 can facilitate evolution of cancer through conferring stemness and EMT as well as induction of cell cycle progression and proliferation.

Diagnostic role of miR-1246 has been vastly appraised in different clinical settings, revealing nearly ideal AUC values, particularly in esophageal, prostate, breast, lung, liver, pancreatobiliary tract and ovarian cancers. The AUC, sensitivity and specificity values obtained for miR-1246 in different cancers are far superior to conventional biomarkers in these cancers. Thus, this miRNA represents an appropriate diagnostic biomarker for neoplastic conditions. Since its levels have been decreased following therapeutic interventions, it has additional advantage in patients’ follow-up. Although miR-1246 can be a putative therapeutic target for cancer, there is no tissue-specific therapeutic approach designed based on miR-1246 until now.

Taken together, miR-1246 is mostly regarded as an oncogenic miRNA in human cancers, albeit some inconsistencies exist for some types of cancers. The interactions of miR-1249 with other types of non-coding RNAs such as lncRNAs and circular RNAs have not been completely assessed. Identification of such interactions has implications in design of diagnostic panels for different cancers.

## Conclusions and Future Perspectives

miR-1246 is an oncogenic miRNA in several tissues. Therapeutic intervention with its expression or methylation pattern can be regarded as a novel modality. However, it is necessary to design tissue-specific therapeutic approaches.
